# HSP70 positively regulates translation by interacting with the IRES and stabilizes the viral structural proteins VP1 and VP3 to facilitate duck hepatitis A virus type 1 replication

**DOI:** 10.1186/s13567-024-01315-9

**Published:** 2024-05-17

**Authors:** Yurui Jiang, Chenxia Xu, Anchun Cheng, Mingshu Wang, Wei Zhang, Xinxin Zhao, Qiao Yang, Ying Wu, Shaqiu Zhang, Bin Tian, Juan Huang, Xumin Ou, Di Sun, Yu He, Zhen Wu, Dekang Zhu, Renyong Jia, Shun Chen, Mafeng Liu

**Affiliations:** 1 Engineering Research Center of Southwest Animal Disease Prevention and Control Technology, Ministry of Education of the People’s Republic of China, Chengdu, 611130 China; 2 Key Laboratory of Animal Disease and Human Health of Sichuan Province, Chengdu, 611130 China; 3 International Joint Research Center for Animal Disease Prevention and Control of Sichuan Province, Chengdu, 611130 China; 4 Institute of Veterinary Medicine and Immunology, Sichuan Agricultural University, Chengdu, 611130 China; 5 Research Center of Avian Disease, College of Veterinary Medicine, Sichuan Agricultural University, Chengdu, 611130 China; 6 Sinopharm Yangzhou VAC Biological Engineering Co., Ltd., Yangzhou, 225100 China

**Keywords:** DHAV-1, HSP70, IRES, VP1, VP3, translation, assembly

## Abstract

The maintenance of viral protein homeostasis depends on the interaction between host cell proteins and viral proteins. As a molecular chaperone, heat shock protein 70 (HSP70) has been shown to play an important role in viral infection. Our results showed that HSP70 can affect translation, replication, assembly, and release during the life cycle of duck hepatitis A virus type 1 (DHAV-1). We demonstrated that HSP70 can regulate viral translation by interacting with the DHAV-1 internal ribosome entry site (IRES). In addition, HSP70 interacts with the viral capsid proteins VP1 and VP3 and promotes their stability by inhibiting proteasomal degradation, thereby facilitating the assembly of DHAV-1 virions. This study demonstrates the specific role of HSP70 in regulating DHAV-1 replication, which are helpful for understanding the pathogenesis of DHAV-1 infection and provide additional information about the role of HSP70 in infection by different kinds of picornaviruses, as well as the interaction between picornaviruses and host cells.

## Introduction

Duck hepatitis A virus (DHAV), a member of the genus *Avihepatovirus* in the family *Picornaviridae*, is a highly lethal virus that acutely infects mainly 1- to 4-week-old ducklings. DHAVs include duck hepatitis A virus type 1 (DHAV-1), duck hepatitis A virus type 2 (DHAV-2) and duck hepatitis A virus type 3 (DHAV-3). Among these viruses, DHAV-1 is the most prevalent and toxic, posing a major economic threat to the duck industry worldwide [1].

DHAV-1 is a typical picornavirus, and the genome of DHAV-1 is a single-stranded positive-sense RNA that contains a single open reading frame and encodes a polyprotein. Infection by DHAV-1 is a complex process that involves multiple steps, including virus adsorption and invasion, translation and replication, and assembly and release [2]. The life cycle of picornaviruses, which include enteroviruses, begins with the interaction of viral particles with specific cytokines [3], such as p-selectin glycoprotein ligand 1 (PSGL-1), heparan sulfate (HS), and annexin A2 (Anx2), which are located on the cell surface [4, 5] and act as attachment receptors to support the binding of viral particles. Then, attachment receptors trap viral particles on the cell surface and deliver them to membrane-free receptors via endocytosis mediated by clathrin [6], caveolin [7] and endotropin-A2 [8]. Next, the binding of uncoating receptors such as scavenger receptor class B member 2 (SCARB2) [6] and human neonatal FC receptor (FcRn) to virions triggers the release of pocket factors and subsequently induces conformational changes in virions [9], resulting in internalization and endosome-dependent uncoating process of virus. As virus RNA enters the cytoplasm, dominant cap-dependent translation which initiated by eukaryotic translation initiation factor 4E (eIF4E), is inhibited. Instead, viruses exploit the internal ribosome entry site (IRES) and IRES trans-acting factors (ITAFs) for translation [10], these proteins are translated into three capsid proteins (VP0, VP1, and VP3) and seven nonstructural proteins (2A, 2B, 2C, 3A, 3B, 3C, and 3D) by a viral protease [11, 12]. Viral RNA replication is initiated when viral proteins accumulate to a certain level. First, viral RNA uses VPg as a primer to mediate the synthesis of negative-stranded RNA to form double-stranded RNA under the action of the viral protein 3D, host factor poly-c binding protein 2 (PCBP2) and polysaccharide binding protein 1 (PABP1) [13]. Next, positive-strand RNA is synthesized in large quantities and is used for the translation or assembly of viral particles. In the final stage of viral infection, precursor P1 proteins are cleaved into VP0, VP1 and VP3 by the 3CD protease. Immediately, VP0, VP1 and VP3 form a protomer, and five protomers then assemble to form a 14S pentamer. Upon the interaction between 2C and the capsid protein VP3 [14] or VP1 [15], the 14S pentamer is recruited to the replication complex, the site of particle assembly, where 12 pentamers are likely to condense around the RNA to form a noninfectious provirion. Finally, infectious virus particles are formed when VP0 is cleaved to produce mature VP2 and VP4 [16].

HSP70s are highly conserved proteins that can be stimulated by pathogens, heat stress, inflammation and other adverse factors. Under stress, HSP70s are expressed at increased levels and play a role in protecting cells by regulating the refolding of misfolded or unfolded proteins and physiological processes such as apoptosis, autophagy and immune responses [17, 18]. Usually, HSP70s act as intracellular guardians, but they can be used by viruses to harm the host organism. Virus produces many viral proteins during its life cycle. To ensure the correct folding of these viral proteins, HSP70s, which are central components of cellular chaperone networks, are used by viruses for viral protein folding. For example, HSP70 can inhibit the proteasomal degradation pathway to stabilize the important viral replication complex components 2C and 3D and promote the replication of EV71 [19]. HSP70 can interact with the poliovirus P1 and extended half-life of P1. Moreover, sucrose density gradient results suggested that the folding and capsid assembly of poliovirus P1 is critically dependent on HSP70 [20]. In addition to using them for viral protein folding, viruses recruit HSP70s, which are involved in cellular processes and host mechanisms, for viral replication, protein translation and viral particle assembly during viral infection, thus increasing the effectiveness of infection [21]. For example, HSP70 can interact with enterovirus 71 (EV71) and promote viral attachment to hose cells through assisting EV71, subsequently enhancing viral endocytosis into host cells and increasing susceptibility to EV71 [22]. HSC70 does not directly interact with the IRES but rather disrupts host cell translation by regulating the activity of the 2A protease, which cleaves eukaryotic translation initiation factor 4 gamma (eIF4G) and activates IRES-mediated translation, thus promoting viral proliferation [23]. Many studies have shown that HSP70 is used by many viruses to promote virus replication in different life cycles. EV71, also belongs to picornaviruses, can use HSP70 to regulate the viral life cycle [19]. However, whether HSP70 regulates DHAV-1 replication is unclear, and studying the effect of HSP70 on DHAV-1 will help us to understand virus‒host interactions and viral pathogenesis.

## Materials and methods

### Cells and viruses

Duck embryo fibroblasts (DEFs) were prepared from 9-day-old duck embryos. After removing the organs of duck embryo, the embryo was minced and then digested with 0.25% trypsin. After centrifugation, DEFs were cultured in Dulbecco’s modified Eagle’s medium (DMEM) supplemented with 10% fetal bovine serum (FBS) (Gibco) at 37 °C in 5% CO2.

The DHAV-1 H strain (GenBank accession number: JQ301467.1) used in this study was provided by the Sichuan Agricultural University Poultry Disease Prevention Research Center.

### Plasmids

pCAGGS-VP1-Flag, pCAGGS-VP3-Flag and pRL-CMV were maintained and provided by the Poultry Disease Research Center of Sichuan Agricultural University.

HA-tagged HSP70 and HA-tagged truncated HSP70 plasmids were constructed as follows. Corresponding cDNAs were amplified by RT‒PCR using total RNA extracted from DEFs as a template, subcloned and inserted into the pCAGGS vector (Table 1).

**Table 1:** **All primer sequences used in this experiment**

Gene	Primer name	Primer sequence (5′-3′)	Refs
β-actin	P1 F	TACGCCAACACGGTGCTG	
	P1 R	GATTCATCATACTCCTGCTTG	
HSP70	P2 F	GGATCTGCTCCTGCTGGATGTC	New
	P2 R	GGAATGGTGGTGTTACGCTTGATG	New
HSP70-HA	P3 F	CATCATTTTGGCAAAGAATTCGCCACCATGTCTGGCAAAGGGCCGGCGAT	New
	P3 R	CACATCATAAGGATAGGTACCATCTACTTCTTCGATGGTCG	New
FLUC	P4 F	GTAGACTTTCATGAAATGGAAGACGCCAAAAACAT	New
	P4 R	TCATGTCTGCTCGAAGCGGCCGCTTACACGGCGATCTTTCCGC	New
IRES	P5 F	AAAATGAACAATAATTCTAGAAGCGTCGTTACACTTGACCT	New
	P5 R	TTTGGCGTCTTCCATTTCATGAAAGTCTACTGGTA	New

“New” refers to primers designed and synthesized in this paper.

CMV-Rluc-Fluc was constructed as follows: the firefly luciferase gene (FLuc) was inserted into the NotI site of pRL-CMV. The dicistronic reporter plasmid CMV-Rluc-IRES-Fluc, which contains the DHAV-1 IRES between Renilla and firefly luciferase, was constructed by inserting an XbaI-DHAV-1-IRES–NotI fragment into CMV-Rluc-Fluc. All the DNA constructs were verified by sequencing.

### Antibodies and reagents

A mouse anti-Flag monoclonal antibody (Cat: M185-3 S) and a mouse anti-HA monoclonal antibody (Cat: M132-3) were purchased from Medical & Biological Laboratories Co., Ltd. A rabbit anti-beta (β)-actin antibody (Cat: 20536-1-AP) was obtained from Proteintech Co., Ltd. An HRP-conjugated goat anti-mouse IgG heavy chain antibody (Cat: AS064) and an HSP70 rabbit pAb antibody (Cat: A7902) were obtained from ABclonal Technology Co., Ltd. A mouse IgG antibody (Cat: A7028) and an HRP-conjugated goat anti-mouse IgG (Cat: A0216) were purchased from Beyotime Co., Ltd. A rabbit anti-VP3 antibody was prepared in our laboratory. An Alexa Fluor™ 568-conjugated goat anti-mouse IgG antibody (Cat: A11004) and an Alexa Fluor™ 488-conjugated goat anti-rabbit IgG antibody (Cat: A11008) were purchased from Thermo Fisher Scientific Co., Ltd. VER155008 (Cat: S7751), MG132 (Cat: S2619), and CHX (Cat: S7418) were purchased from Selleckchem. All drugs used in this study were dissolved in 0.1% DMSO.

### Biotinylated RNA pulldown assay

For the biotinylated RNA pulldown assay, we used the Pierce™ RNA 3’End Desthiobiotinylation Kit (Cat: 20163) and Pierce™ Magnetic RNA–Protein Pull-Down Kit (Cat: 20164) (Thermo Fisher Scientific Co., Ltd.) according to the manufacturer’s instructions. cDNA containing the DHAV-1 IRES and the T7 promoter was synthesized using specific primers, transcribed in vitro using a TranscriptAid T7 High Yield Transcription Kit (Cat: K0441) (Thermo Fisher Scientific) and purified. Then, T4 RNA ligase was used to attach a single biotinylated nucleotide to the 3´ terminus of the RNA strand. Then, 50 µL of streptavidin magnetic beads were added to 50 pmol of labeled RNA and incubated for 30 min at 37 °C. Then, the RNA-magnetic bead complex was added to the cell lysates for binding to RNA-binding proteins. Finally, the magnetic beads were washed three times, and the supernatant was collected and incubated with elution buffer for Western blot analysis.

### RNA–protein coimmunoprecipitation

For RNA–protein coimmunoprecipitation, DEFs were transfected with appropriate plasmids for 24 h and then infected with DHAV-1 for 12 h. The cells were washed in PBS, lysed with RIPA buffer (Cat: P0013B) (Beyotime Biotechnology) for 30 min and centrifuged at 12 000 × *g* for 10 min at 4 °C. 6 μL mouse anti-HA monoclonal antibody or 6 μL control mouse IgG was added at a ratio of 1:100 and incubated at 4 °C for 24 h. Then, BeyoMag™ Protein A + G (Cat: P2108) (Beyotime Biotechnology) was added at a ratio of 1:25, and the mixture was incubated at 4 °C for 4 h. The complex was washed three times with DEPC water, and total RNA was extracted and reverse transcribed into cDNA. Finally, PCR analysis was performed by using specific primers for the DHAV-1 IRES.

### Cell viability assay

When the cells reached 80% confluence in 96-well plates, they were treated with the indicated inhibitors at different concentrations or transfected with siRNAs at 37 °C for 36 h. Cell viability was determined by a cell counting kit-8 (Cat: C0037) (Beyotime Biotechnology) according to the manufacturer’s instructions. CCK8 solution was added to each well, and the plates were incubated at 37 °C for 1 h. The OD_450_ was measured using a microplate reader.

### RNA extraction and qPCR

Total RNA was extracted from the samples using RNAiso Plus Reagent (Cat: 9109) (TaKaRa) according to the manufacturer’s instructions. Total RNA was reverse transcribed into cDNA using the PrimeScript™ RT Reagent Kit (Perfect Real Time) (Cat: RR047A) (TaKaRa, Japan). HSP70 and β-actin transcript levels were quantified through quantitative real-time PCR (RT‒qPCR) using a SYBR® Premix Ex Taq™ II (Tli RNaseH Plus) Kit (Cat: RR420A) (Takara). Relative mRNA expression levels were normalized to that of the housekeeping gene β-actin. The viral copy number in cells was determined according with one-step TaqMan fluorescent quantitative RT‒PCR in our laboratory [24].

### Virus titration

The degree of virus infectivity was determined by endpoint dilution. Serially diluted samples were used to infect the indicated cells in 48-well plates, the cytopathic effect of DEFs were observed under microscope and the TCID_50_ was calculated using the Reed–Muench method.

### Dual‑luciferase reporter assay

DEFs in which HSP70 was knocked down or overexpressed as described above were transfected with CMV-Rluc-IRES-Fluc or CMV-Rluc-Fluc. Samples were collected 24 h after transfection, and firefly and Renilla luciferase activities were analyzed using a TransDetect® Double-Luciferase Reporter Assay Kit (Cat: FR201-02-V2) (TransGen Biotech). The ratio of FLuc expression to RLuc expression represents relative DHAV-1-IRES activity.

### Indirect immunofluorescence assay

DEFs were grown on coverslips and transfected for 24 h. After transfection, the cells were washed three times with PBS, fixed with 4% paraformaldehyde at 37 °C for 3 h, permeabilized with 0.25% Triton X-100 at 4 °C for 30 min, and fixed with 5% BSA at 37 °C for 3 h. Rabbit anti-HA (1:1000) and mouse anti-Flag (1:1000) antibodies were used as primary antibodies and incubated overnight at 4 °C. Then, the cells were incubated with Alexa Fluor™568-conjugated goat anti-mouse IgG (1:1000) and Alexa Fluor™ 488-conjugated goat anti-rabbit IgG (1:1000) secondary antibodies at room temperature for 1 h. The nuclei were stained with DAPI (1:1000; D9542, Sigma) at room temperature for 15 min and visualized under an inverted fluorescence microscope.

### Western blot analysis

The supernatant was discarded from the transfected cells, and the cells were washed with cold PBS and lysed with RIPA buffer (Beyotime Biotechnology). The proteins were separated by SDS‒PAGE and subsequently transferred to PVDF membranes (Bio-Rad). The membranes were blocked at 37 °C for 3 h with 5% skim milk powder, incubated with primary antibodies overnight, washed with Tris-buffered saline-Tween (TBST) three times, and incubated with HRP-labeled goat anti-rabbit IgG or HRP-labeled goat anti-mouse IgG at 37 °C for 1 h. The protein bands were visualized using enhanced chemiluminescence (ECL) (Bio-Rad) detection reagent.

### Co‑immunoprecipitation

At 24 h post-transfection, the cells were washed in PBS, lysed with RIPA buffer (Beyotime Biotechnology) for 30 min and centrifuged at 12 000 × *g* for 10 min at 4 °C. 4 μL mouse anti-HA or 4 μL mouse anti-Flag antibodies were added at a ratio of 1:100 and incubated at 4 °C for 24 h. Then, BeyoMag™ Protein A + G (Beyotime Biotechnology) was added at a ratio of 1:25, and the mixture was incubated at 37 °C for 1 h. After washing four times with TBS, the supernatant was discarded, 40 µL of PBS was added, and 10 µL of 5 × loading buffer was added to the eluted samples, which were subjected to Western blot analysis.

### Gene knockdown by siRNA

siRNAs targeting the HSP70 gene and negative control siRNA were synthesized by GenePharma. When the cells reached 80% confluence, they were transfected with siRNAs using Lipofectamine™ 2000 (Cat: 11668019) (Thermo Fisher Scientific) according to the manufacturer's instructions. The sequences of the siRNAs are as follows: siRNA-772, 5′ -GGUGCCUGCUUACUUCAAUTT; siRNA-1000, 5′ -GCAUGGUGAACCACUUUGUTT; siRNA-1777, 5′ -CGGGUAAGGAGAACAAGAUTT; siRNA-1948, 5′ -CGGUGGAGGAUGAUAAACUTT.

### Statistical analysis

In this paper, the data are presented as group means and standard deviations (SDs) and were analyzed by Student’s *t* test or two-way analysis of variance (ANOVA) using GraphPad Prism software version 8. Results with *P ≤ 0.05 were considered statistically significant.

## Results

### Effect of the HSP70 inhibitor VER155008 on DHAV-1

**Figure 1:**
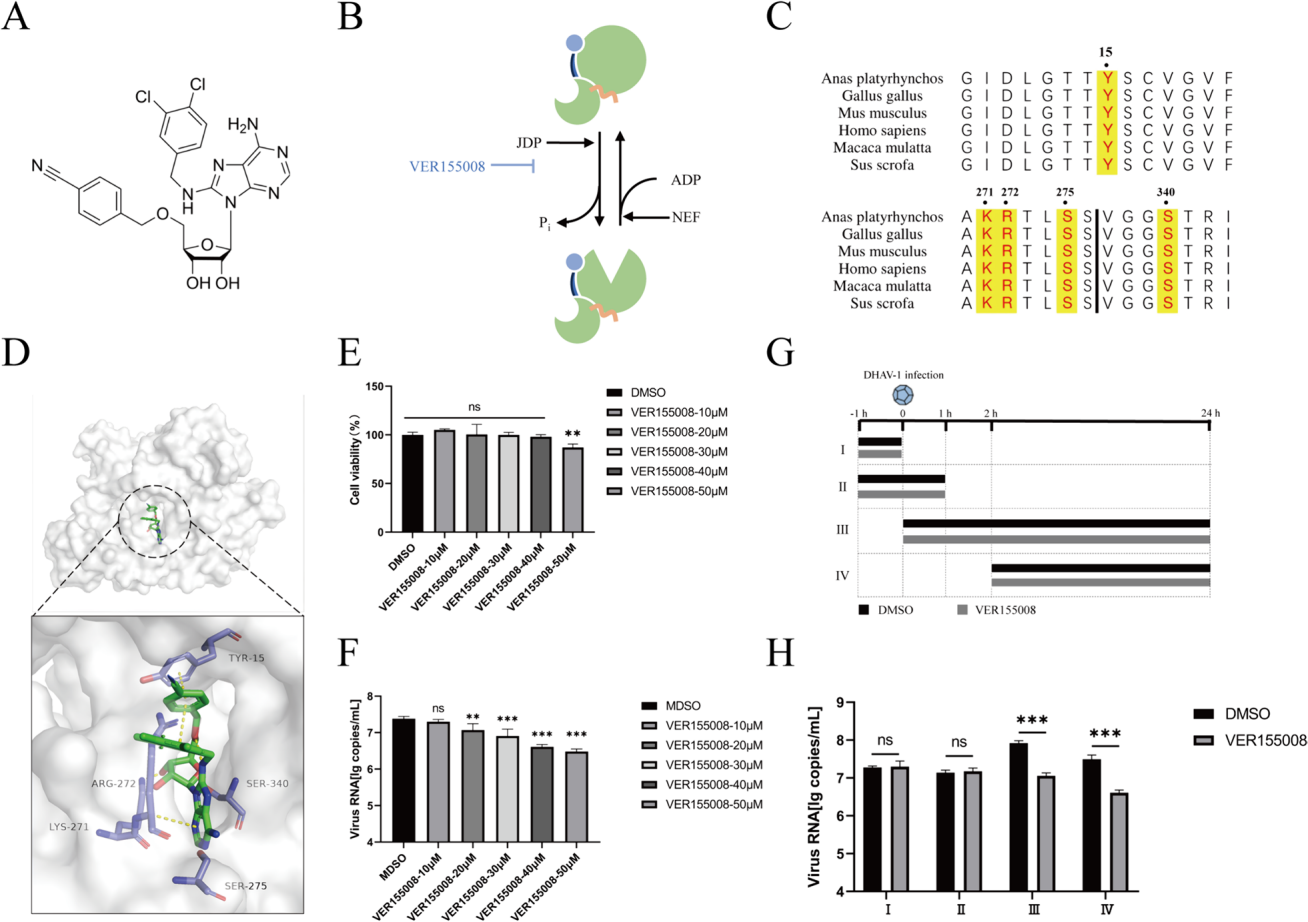
**HSP70 is crucial for DHAV-1 replication in DEFs. A** The chemical formula for VER155008. **B** VER15508 blocks ATP binding to HSP70. **C** Sequence alignment of human HSP70 with HSP70 of other species. The key sites involved in the interaction of HSP70 with VER155008 are marked in red. **D** Molecular docking of HSP70 and VER155008. **E** DEFs were treated with different concentrations of VER155008, and cell viability was analyzed via a CCK-8 kit after 36 h. **F** Different concentrations of the VER155008 inhibitor were applied, and DEFs were infected with DHAV-1 at the same time. The cells were harvested at 36 h, and the viral copy number was quantified via one-step TaqMan fluorescent quantitative RT‒PCR. **G**, **H** Time course of the drug treatment experiment. VER155008 was applied at different times. The viral copy number was quantified. **, *P* < 0.01; ***, *P* < 0.001; ns, not significant.

To investigate the role of HSP70 in DHAV-1 infection, we inhibited HSP70 protein function using VER155008, an HSP70-specific inhibitor that competitively binds HSP70 to ATP [25, 26] (Figures 1A and B). To ensure the generality of the inhibitor, we assessed its ability to inhibit the function of *Anas platyrhynchos* HSP70 by sequence alignment and molecular docking analysis (Figures 1C and D). DHAV-1-infected DEFs were treated with VER155008 at different concentrations. VER155008 at a concentration of 40 μM had the optimal inhibitory effect on DHAV-1 infection without affecting cell viability (Figures 1E and F). Then, VER155008 was added to DHAV-1-infected cells from − 1 to 0 h (I), − 1 to 1 h (II), 0 to 24 h (III), or 2 to 24 h (IV), corresponding roughly the adsorption, invasion, translation and replication of DHAV-1 [19, 27], respectively, to clarify which stage of the DHAV-1 life cycle requires HSP70 (Figure 1G). Compared with those in the DMSO group, the viral copy numbers in VER155008-treated groups I and II were not different, while the viral copy numbers in group III and group IV were significantly lower than those in the control group (Figure 1H).

### HSP70 facilitates multiple steps in the DHAV-1 life cycle

**Figure 2:**
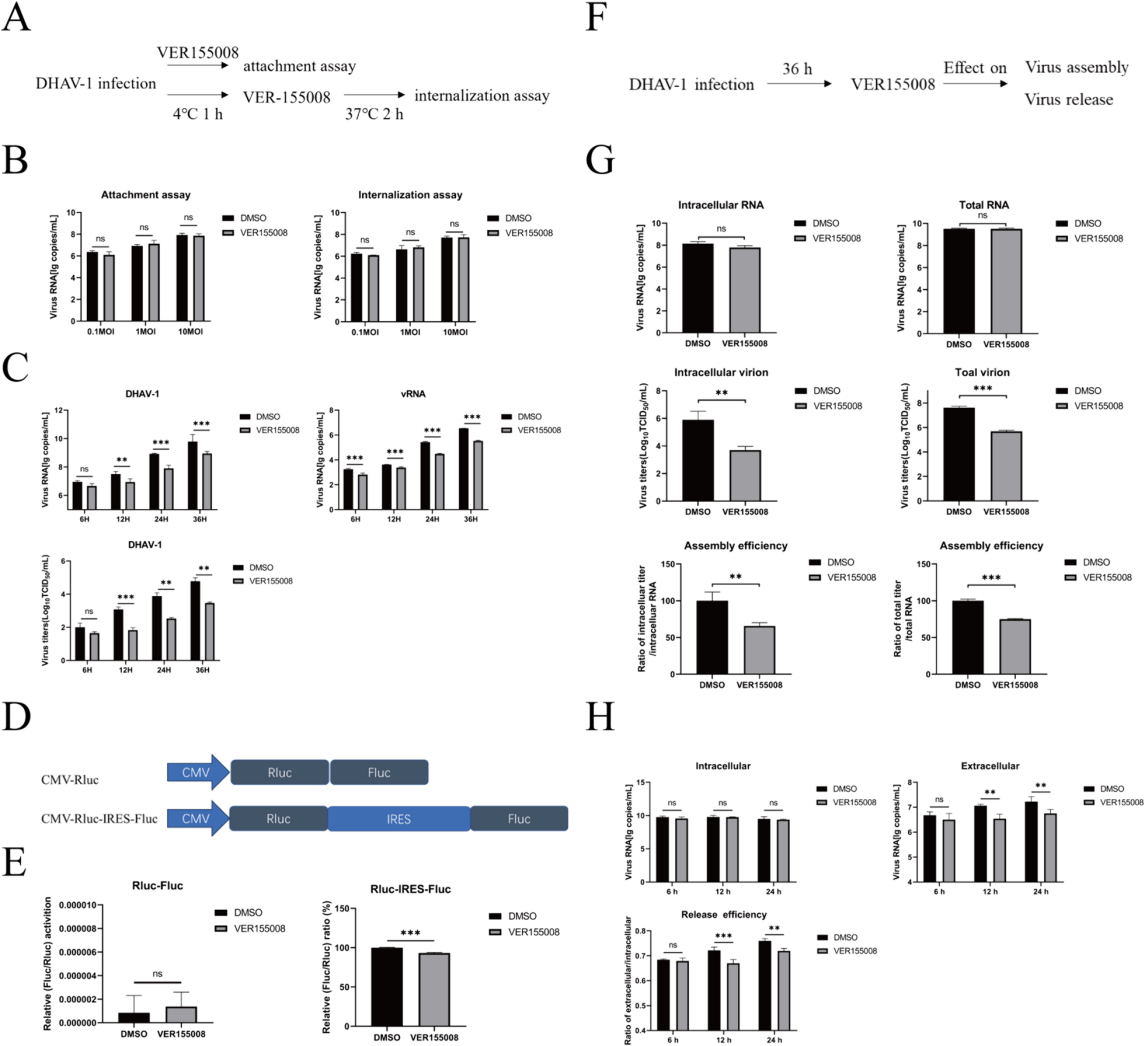
**HSP70 affects multiple steps of the DHAV-1 life cycle.** **A**, **B** Attachment assay. DEFs were precooled at 4 °C for 30 min, after which DMED was replaced with VER155008, DMSO or DHAV-1 (0.1 MOI, 1 MOI, or 10 MOI). After incubation at 4 °C for 4 h, the cells were washed three times with cold PBS. For the internalization assay, After the attachment of DHAV-1, the medium was replaced with VER155008 or DMSO, and the cells were incubated at 37 °C for 2 h and then washed three times. One-step TaqMan fluorescent quantitative RT‒PCR was used to measure the viral copy number. **C** DEFs were incubated with VER155008, DMSO, or DHAV-1, and the cells were harvested at 6 h, 12 h, 24 h, or 36 h. The total vial negative viral copy number of DHAV-1 were analyzed via RT‒PCR. Viral production was analyzed by TCID_50_. **D**, **E** Schematic diagram of the dual-luciferase plasmids. DEFs were transfected with Rluc-IRES-Fluc and incubated with VER155008 or DMSO. After 24 h, the cells were harvested for dual-luciferase assays. **F**, **G**, **H** DEFs were infected with DHAV-1. After 36 h, VER155008 or DMSO was added to the cells, and the cells and supernatants were harvested at 6 h, 12 h, and 24 h. The extracellular RNA/intracellular RNA ratio represents viral release efficiency. Supernatants and cells were harvested at 12 h. The viral TCID_50_/viral RNA ratio represents viral assembly efficiency. **, *P* < 0.01; ***, *P* < 0.001; ns, not significant.

To further prove the specific role of HSP70 in DHAV-1 infection, various experiments were carried out to explore the effect of HSP70 on the life cycle of the virus. DEFs treated with VER155008 were inoculated with DHAV-1 and incubated at 4 °C for virus attachment. DHAV-1 was added and incubated at 4 °C for virus adsorption, and VER155008 was added 1 h later, after which the mixture was incubated at 37 °C to allow virus entry (Figure 2A). Compared with DMSO, VER155008 did not affect DHAV-1 adsorption or invasion at different concentrations (Figure 2B). To study the effect of virus infection on replication, DEFs were infected with the virus, after which the total and negative-strand copy numbers and the TCID_50_ were determined. The addition of VER155008 decreased the total and negative-strand copy numbers and viral titer, indicating that VER155008 can inhibit DHAV-1 replication (Figure 2C). A bicistronic luciferase plasmid containing the cap-dependent Renilla luciferase gene (Rluc) and the mediated firefly luciferase gene (Fluc) under the control of the DHAV-1 IRES was constructed (Figure 2D) to investigate whether HSP70 is a driver of DHAV-1 IRES-mediated translation. The effect of HSP70 on DHAV-1 IRES-mediated translation was determined using CMV-Rluc-IRES-Fluc, and VER155008 was added after transfection. VER155008 inhibited the activity of the IRES, indicating that HSP70 promotes viral IRES-mediated translation (Figure 2E). VER155008 was added to DEFs after 36 h of virus infection. The viral copy number in the supernatant/the viral copy number in the cell represents virus release efficiency, and the viral TCID_50_/viral copy number represents viral assembly efficiency (Figure 2F). VER155008 strongly inhibited the assembly of the virus (Figure 2G), and the decrease in release efficiency may have been attributable to the effect of VER155008 on assembly (Figure 2H). We conclude that HSP70 does not affect DHAV-1 adsorption or invasion but participates in viral translation, replication and assembly.

### DHAV-1 induces the expression of HSP70

**Figure 3:**
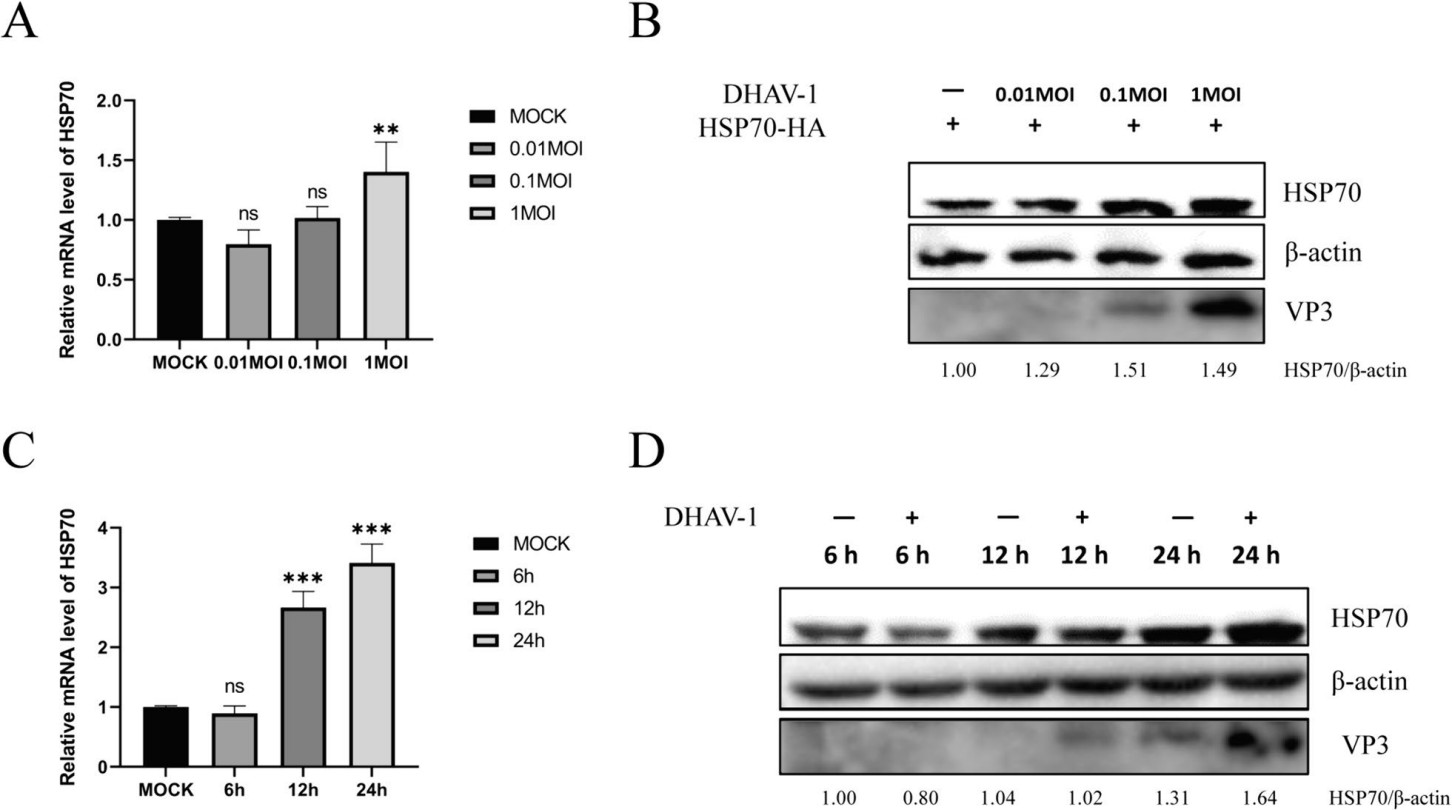
**Cellular HSP70 mRNA and protein expression levels in DEFs infected with DHAV-1. A**, **B** DEFs were infected with DHAV-1 (0.01 MOI, 0.1 MOI, or 1 MOI). Cells were collected to analyze the mRNA expression of HSP70 by quantitative RT‒PCR and the protein expression of HSP70 by Western blotting. **C**, **D** DEFs were infected with DHAV-1 at an MOI of 0.1 and harvested at 6 h, 12 h, and 24 h, after which the mRNA expression and protein expression of HSP70 were analyzed. **, *P* < 0.01; ***, *P* < 0.001; ns, not significant.

As a stressor, viral infection can promote the expression of heat shock proteins, and many induced heat shock proteins can be used by the virus to increase its own infectivity. To study the effect of DHAV-1 on the expression of HSP70, cell samples were collected after inoculation with DHAV-1 for RT‒PCR and Western blotting. The results showed that the levels of HSP70 transcription and protein expression increased as the virus titer increased (Figures 3A and B). With increasing time, the level of HSP70 transcription and protein expression also increased (Figures 3C and D).

### Effects of HSP70 overexpression and knockdown on DHAV-1 replication

**Figure 4:**
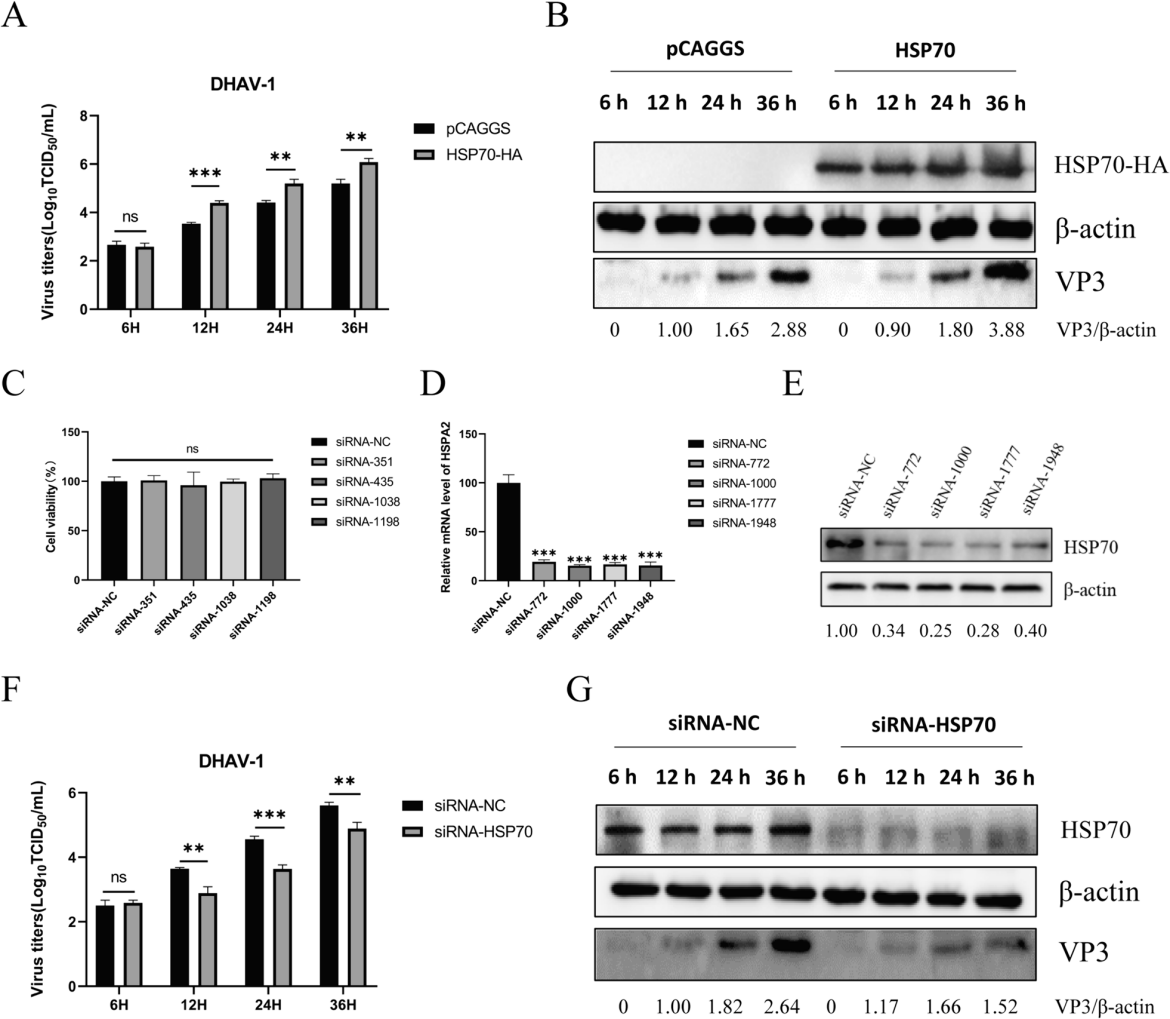
**Effect of HSP70 overexpression or knockdown on DHAV-1 replication.** **A**, **B** DEFs were transfected with pCAGGS-HSP70-HA or pCAGGS. After transfection, the cells were infected with DHAV-1 and harvested at 6 h, 12 h, 24 h, and 36 h. Virus production in the cells was assessed by measuring the TCID_50_, which involved measuring viral protein expression by Western blotting. **C**, **D**, **E** DEFs were transfected with siRNA-NC or siRNAs targeting the HSP70 gene. After 36 h, cell viability was analyzed with a CCK-8 kit, and the mRNA expression and protein expression of HSP70 were analyzed via RT‒PCR and Western blotting, respectively. **F**, **G** DEFs were transfected with siRNA-NC or siRNA-HSP70. After transfection, the cells were infected with DHAV-1 and harvested at 6 h, 12 h, 24 h, and 36 h. Virus production and viral protein expression were analyzed. **, *P* < 0.01; ***, *P* < 0.001; ns, not significant.

In our study, ectopic expression of HSP70 promoted viral replication (Figures 4A and B). Transfection of siRNA to knock down HSP70 did not affect cell viability (Figure 4C) or reduce the transcription or protein levels of HSP70 (Figures 4E and F). After siRNA transfection, cells were inoculated with DHAV-1, and knockdown of HSP70 reduced the viral copy number and viral titer (Figures 4G, F and I). These results suggest that HSP70 is essential for DHAV-1 infection.

### HSP70 interacts with the IRES and regulates DHAV-1 translation

**Figure 5:**
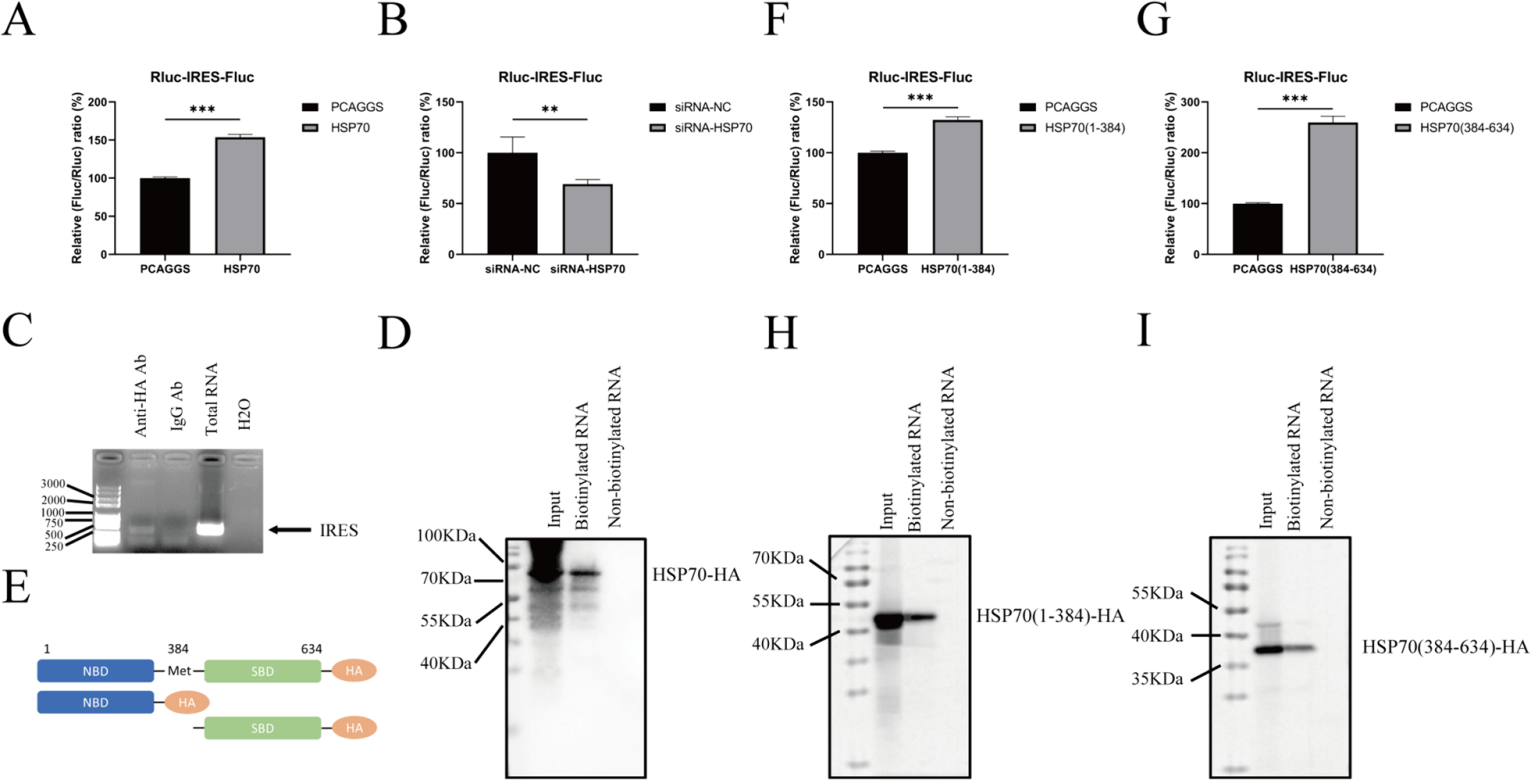
**HSP70 regulates DHAV-1 translation through interaction with the IRES. A** DEFs were co-transfected with Rluc-IRES-Fluc and HSP70-HA. After 24 h, the samples were harvested for dual-luciferase assays. **B** DEFs were transfected with siRNA-HSP70 or siRNA-NC and then transfected with Rluc-IRES-Fluc after 36 h. Samples were collected at 18 h post-transfection, and luciferase activity was measured. **C** DEFs were transfected with HSP70-HA, and the cells were infected with DHAV-1 at 24 h post-transfection. The cell lysates were collected, and a mouse anti-HA antibody or mouse anti-IgG was added. The pulled down RNAs were extracted and amplified via RT‒PCR using DHAV-1 IRES primers. **D** DEFs were transfected with HSP70-HA. After 24 h, the cell lysates were incubated with biotinylated or nonbiotinylated IRES, RNA-binding proteins were collected, and their expression was analyzed via Western blotting. **E** Schematic diagram of Hsp70 truncation mutants. **F**, **G** HA-tagged truncated forms of HSP70, i.e., HSP70(1-384)-HA and HSP70(384-634)-HA, were co-transfected into cells with Rluc-IRES-Fluc, and luciferase activity was measured. **H**, **I** HA-tagged truncated forms of HSP70 were incubated with biotinylated or nonbiotinylated IRES and analyzed via an RNA pulldown assay. **, *P* < 0.01; ***, *P* < 0.001.

DHAV-1 translation is mediated mainly by the IRES in the 5'UTR. We next investigated whether HSP70 is a driver of DHAV-1 IRES-mediated translation. The expression of HSP70 promoted viral translation (Figure 5A), while the knockdown of HSP70 inhibited translation (Figure 5B). The results show that HSP70 promotes DHAV-1 IRES-driven translation.

We next aimed to demonstrate that HSP70 interacts with the IRES of DHAV-1 in infected DEFs. An anti-HA monoclonal antibody was used for immunoprecipitation of virus-infected DEF lysates, and normal mouse IgG served as a negative control. Then, the immune complex was isolated and amplified via RT‒PCR using IRES-specific primers. A cDNA band of the expected size (300 bp) was observed in the anti-HA antibody group but not in the mouse IgG group. The data indicate that HSP70 can specifically interact with the IRES during DHAV-1 infection (Figure 5C).

To explore whether HSP70 promotes viral translation by interacting with the IRES, an RNA pulldown assay was performed with a biotinylated form of the DHAV-1 IRES and cell lysates. HSP70 bound to biotinylated RNA but not to nonbiotinylated RNA, showing that HSP70 interacts with the DHAV-1 IRES specifically (Figure 5D).

HSP70 consists of a nucleotide binding domain (NBD) and a substrate binding domain (SBD). To confirm which specific functional domains of HSP70 affect DHAV-1 translation, cells were co-transfected with CMV-Rluc-IRES-Fluc and truncated HSP70 plasmids (Figure 5E) for luciferase assays. The results showed that the truncation of different functional domains of the SBD and NBD promoted the translation of viral proteins (Figures 5F and G). Then, an RNA pulldown assay was performed, and the truncated plasmids were found to directly interact with the DHAV-1 IRES (Figures 5H and I). This suggests that both the NBD and SBD of HSP70 may affect IRES-mediated translation by interacting with the IRES.

### Interaction of HSP70 and its different functional domains with the structural proteins VP1 and VP3

**Figure 6:**
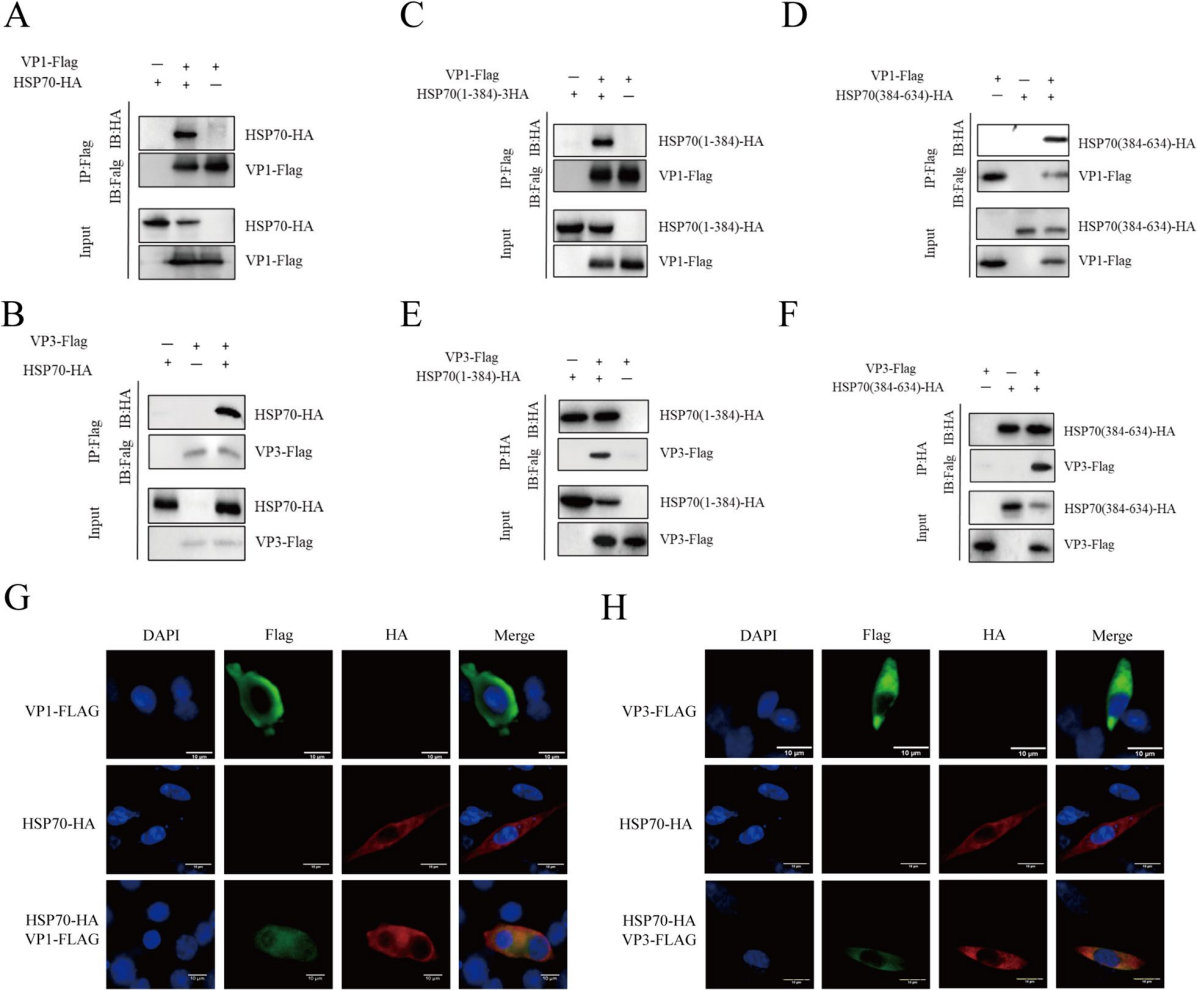
**HSP70 and its different functional domains interact with the structural proteins VP1 and VP3. A**, **B** Co-IP analysis of exogenous HSP70, VP1, and VP3. DEFs were co-transfected with HSP70, VP1-Flag, and VP3-Flag. After 24 h, the cell lysates were incubated with a mouse anti-HA or mouse anti-Flag antibody, and anti-HA and anti-Flag antibodies were subsequently used for immunoblotting. **C, D, E, F** Co-IP analysis of exogenous HA-tagged truncated HSP70, VP1, and VP3. Cell lysates were used for coimmunoprecipitation. **G, H** Colocalization of HSP70 with VP1 and VP3. HSP70, VP1-Flag, and VP3-Flag were transfected into DEFs. An indirect immunofluorescence assay was performed with mouse anti-HA and mouse anti-Flag antibodies and DAPI.

HSP70 affects the assembly of DHAV-1, which depends on the functions of VP1 and VP3. VP1 and VP3 were co-transfected into DEFs with HSP70. Coimmunoprecipitation revealed interactions between HSP70 and VP1 and between HSP70 and VP3 (Figures 6A and B). HSP70 colocalized with VP1 and VP3 in the cytoplasm, as shown by indirect immunofluorescence, which further confirmed the interaction between HSP70 and VP1 and between HSP70 and VP3 (Figures 6G and H). To determine which domain of HSP70 interact with these viral proteins, plasmids containing truncated forms of HSP70 were co-transfected into cells with VP1 and VP3. Our results showed that the NBD and SBD domain of HSP70 interact with VP1 and VP3 (Figures 6C–F).

### HSP70 promotes the stability of the structural proteins VP1 and VP3 by inhibiting proteasomal degradation

**Figure 7:**
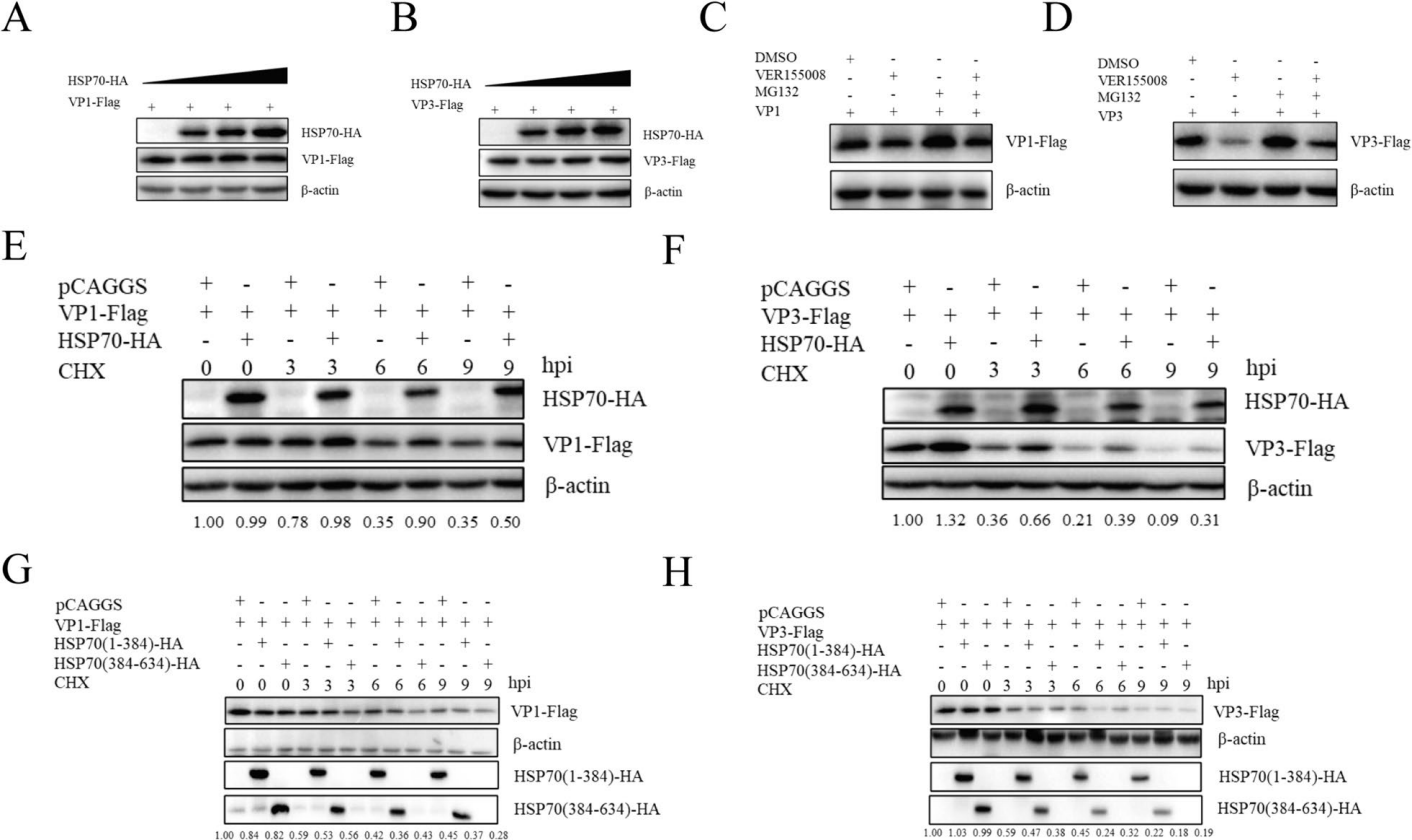
**HSP70 regulates the proteasomal degradation of the structural proteins VP1 and VP3. A, B** Cells were co-transfected with different concentrations of HSP70-HA, VP1-Flag, and VP3-Flag, and protein expression was analyzed by Western blotting at 24 h post-transfection. **C, D** DEFs were transfected with VP1-Flag or VP3-Flag and incubated with VER155008 or DMSO. After 24 h, MG132 was added to the medium, and after 12 h, protein expression in the cell lysates was analyzed by Western blotting. **E, F** DEFs were co-transfected with HSP70-HA, VP1-Flag, and VP3-Flag, and the cells were treated with CHX (100 mg/μL) after 24 h. The cell lysates were harvested at 0, 3, 6, and 9 h, after which protein expression was measured via Western blotting. **G, H** DEFs were co-transfected with HSP70(1-384)-HA or HSP70(384-634)-HA and with VP1-Flag or VP3-Flag and treated with CHX. VP1 and VP3 protein expression levels were analyzed via Western blotting.

Different doses of the HSP70 plasmids were co-transfected with VP1 and VP3, and Western blot analysis showed that HSP70 did not affect the expression levels of VP1 or VP3, suggesting that HSP70 does not interact with VP1 or VP3 in a dose-dependent manner (Figures 7A and B).

HSP70 is a heat shock protein that acts as a chaperone to promote protein folding and assist in maintaining the correct conformation. Previous results showed that HSP70 promotes the assembly of DHAV-1, and it was speculated that HSP70 can affect the assembly of DHAV-1 by ensuring the correct folding of capsid proteins. Therefore, we hypothesized that HSP70 might regulate the stability of VP1 and VP3. To confirm our hypothesis, VP1 and VP3 were transfected into DEFs, and DMSO or VER155008 was added. The protein expression levels of VP1 and VP3 were significantly decreased in the inhibitor group compared with the DMSO control group (Figures 7C and D). The half-lives of VP1 and VP3 in DEFs treated with cycloheximide (CHX) were subsequently analyzed via Western blotting. The results showed that overexpression of HSP70 significantly prolonged the accumulation of VP1 and VP3 (Figures 7E and F). As shown in Figures 7C and D, after MG132 was used to inhibit the protein degradation pathway, it was found that MG132 effectively reversed the decreases in the protein levels of VP1 and VP3 induced by the inhibitor. These results suggest that HSP70 can regulate the proteasome pathway to maintain the stability of VP1 and VP3.

The two domains of HSP70 interact with the viral proteins VP1 and VP3. To explore which functional domain of HSP70 stabilizes the structural proteins VP1 and VP3, truncated HSP70, VP1, and VP3 were co-transfected into DEFs treated with CHX, and the expression of the VP1 and VP proteins was measured. Overexpression of truncated HSP70 did not stabilize VP1 or VP3, suggesting that the synergistic action of the two functional domains of HSP70 is required for the protein to exert its function (Figures 7G and H).

## Discussion

RNA viruses rely on host mechanisms to support the viral life cycle because of their small genomes. During viral infection, large amounts of viral proteins are synthesized rapidly, so the correct folding of these viral proteins is a limiting step in virus infection [28]. It has been reported that HSP70 is a key factor used by many viruses, and the addition of HSP70 inhibitors inhibits the replication of viruses such as EV71 of the *Picornaviridae* family [19], Zika virus [29] and dengue virus [27] of the *Flaviviridae* family, porcine reproductive and respiratory syndrome virus (PRRSV) of the *Arteriviridae* family [30], and rabies virus of the *Rhabdoviridae* family [31]. Here, we investigated the role of HSP70 in multiple steps in the life cycle of DHAV-1. Our results showed that HSP70 was involved in the translation, replication, assembly, and release of DHAV-1, highlighting the important role of HSP70 in DHAV-1 infection.

It is a common that HSP70 is involved in viral proliferation. A large amount of studies indicates that HSP70 chaperones are involved in different stages of viral life cycle. The life cycle of the virus begins with the binding of the virus and cell surface receptors. Previous studies have reported that EV71 [22], Zika virus [29, 32], dengue virus [27], PEDV [33], PRRSV [34], and TGEV [35] use HSP70 located on the cell surface to assist in virus adsorption and invasion. Therefore, we performed a drug treatment experiment [36] to explore the role of HSP70 in the DHAV-1 life cycle. Unlike EV71 and Zika that use HSP70 to participate in the whole life cycle, our results showed that HSP70 was involved mainly in the post-entry step of HSP70 replication but was not involved in attachment or entry. It has also been reported that TUMV of the *Flaviviridae* family does not affect virus adsorption but participates in virus replication [37], which is similar to DHAV-1, suggesting that different viruses may utilize HSP70 in different life cycle processes. We suspect that this difference might be related to the type of cells, the expression and distribution of HSP70 in cells, and the characteristics of virus itself. Further analysis revealed that HSP70 affected the translation, replication, assembly, and release of DHAV-1. This study focused on the effects of HSP70 on DHAV-1 translation and assembly.

After viral internalization into the cell, cap-dependent translation is inactivated, and the virus initiates IRES-dependent translation and recruits host proteins to assist translation by binding to the 5'UTR. HSP70 has been reported to be involved in the translation of picornaviruses by promoting the production, folding, and transport of ITAFs. HSC70 promotes eIF4G cleavage and regulates IRES activity by interacting with the viral protein 2Aof EV71 [23]. HSPA6 increases EV71 IRES activity by acting on IATFs rather than viral proteins [38]. It has been reported that HSP70 has potential RNA binding activity and is associated with HCV 3'-NTR [39]. However, HSP70 directly binds to the 5'UTR to regulate IRES activity in picornaviruses has not been reported. Therefore, we investigated the specific mechanisms by which HSP70 affects translation. First, a luciferase assay showed that HSP70 positively regulated the activity of the IRES. Next, we determined the relationship between HSP70 and the IRES during DHAV-1 infection by RNA‒protein coimmunoprecipitation. Finally, an RNA pulldown assay showed that HSP70 could interact with the IRES, suggesting that HSP70 could regulate translation by directly binding to the IRES. This result is inconsistent with what was reported in other articles, and we suspect that this may be due to the unique type 4 structure of the DHAV-1 IRES.

In the present study, inhibition of HSP70 function significantly reduced the RNA level of DHAV-1, suggesting that HSP70 is a host factor required for virus replication, which is consistent with the findings of previous reports. HSP70s interact directly with viral polymerases to enhance viral replication, or they can promote the formation of viral replication complexes or maintain the stability of complex proteins. For example, the HSP70 protein can interact with the antioxidant response element (ARE) attached to the poly-A tail of the coxsackievirus B3 (CVB3) to stabilize its genome and promote viral replication [40]. EV71 induces cytoplasmic redistribution of GRP78 to promote viral replication [41]. HSP70 also promotes the replication of EV71 by inhibiting the proteasomal degradation pathway to stabilize 2C and 3D, important proteins in the viral replication complex. HSP70 is involved in the formation of the Kaposi’s sarcoma-associated herpesvirus (KSHV) replication and transcription compartments [42]. Whether HSP70 of DHAV-1, like other picornaviruses, promotes DHAV-1 replication by affecting viral polymerase and replication complex is unclear and needs further study.

The morphogenesis of picornaviruses includes P1 protein processing, capsid assembly, RNA packaging and virus maturation. However, the interaction between capsid proteins and other viral proteins is indispensable for the morphogenesis of picornaviruses. It has been reported that the capsid proteins VP1 and VP3 can interact with 2C to facilitate virus assembly. In addition, host factors also play an important role in virus morphogenesis. For example, HSP70 can interact with the P1 protein of PV and CVB3 and prolong the half-life of the viruses. Sucrose gradient centrifugation results have also suggested that HSP70 is involved in the folding and processing of the viral protein P1. In EV71, HSP70 is also involved in regulating the late stages of the assembly process. In addition to its effect on picornaviruses, HSP70 also affects the assembly of other viruses. Heat shock protein inhibitors block the assembly of hepatitis C virus (HCV) [43]. HSC70 also binds to the NS5A protein of HCV and affects viral particle production [44]. According to our results, HSP70 can interact with VP1 and VP3 and colocalize with them in the cytoplasm. MG132 inhibits the degradation of the VP1 and VP3 proteins in DEFs when HSP70 inhibitors are added, and the overexpression of HSP70 prolongs the half-lives of VP1 and VP3 and increases the stability of these viral proteins [45], suggesting that HSP70 can interact with the capsid proteins VP1 and VP3 to protect them from degradation, thus affecting the formation of mature virions.

In conclusion, our study indicates that HSP70 is involved in the regulation of DHAV-1 translation and promotes the production of virions by stabilizing the structural proteins VP1 and VP3. These results may help us to understand the mechanism of DHAV-1 infection and provide insights for the development of antiviral drugs targeting DHAV-1.

## Data Availability

The data that support the findings of this study are available from the corresponding author, M W, upon reasonable request.
